# Successful Direct Acting Antiviral Therapy in Chronic Hepatitis C Normalizes IFNγ and IL2 Production in T Cells Together with TLR8 Expression and Functionality in Peripheral Blood Mononuclear Cells

**DOI:** 10.3390/v13040635

**Published:** 2021-04-07

**Authors:** María Teresa Arias-Loste, Joaquín Cabezas, Susana Llerena, Paula Iruzubieta, David San-Segundo, David Merino, Antonio Cuadrado, José Pedro Vaqué, Marcos López-Hoyos, Javier Crespo

**Affiliations:** 1 Gastroenterology and Hepatology Department, Marqués de Valdecilla University Hospital, 39008 Santander, Spain; mteresa.arias@scsalud.es (M.T.A.-L.); joaquin.cabezas@scsalud.es (J.C.); susana.llerena@scsalud.es (S.L.); paula.iruzubieta@scsalud.es (P.I.); antonio.cuadrado@scsalud.es (A.C.); 2 Group of Clinical and Translational Research in Digestive Diseases (IDIVAL), 39008 Santander, Spain; vaquej@unican.es; 3 Immunology Department, Marqués de Valdecilla University Hospital, 39008 Santander, Spain; david.sansegundo@scsalud.es; 4 Transplant and Autoimmunity Group, Research Institute Marqués de Valdecilla (IDIVAL), 39008 Santander, Spain; 5 Flow Cytometry and Cell Isolation Unit (IDIVAL), 39008 Santander, Spain; david.merino@hotmail.com; 6 Molecular Biology Department, University of Cantabria, 39008 Santander, Spain

**Keywords:** chronic hepatitis C, toll-like receptors, direct acting antivirals

## Abstract

Chronic hepatitis C infection (HCV) activates a systemic cell-mediated immune response characterized by the production of IFNγ and an innate immune response addressed by the activation of TLR signaling. We aimed to investigate whether HCV eradication by direct acting antivirals (DAA) leads to a recovery in cell-mediated immune response and TLR expression and functionality. Blood samples were obtained in HCV infected patients before DAA treatment and at week +48 after the end of treatment. Results were compared to healthy controls. Cell surface expression of TLR8 was assessed on peripheral blood mononuclear cells (PBMCs) by flow cytometry. Freshly isolated PBMCs were cultured with specific TLR8 agonists and intracellular production of cytokines was determined by flow-cytometry after ex vivo TLR8 activation with ssRNA 40. Production of IFNγ, IL2 and IL17 was assessed by flow cytometry in T cells after polyclonal activation. Included were 50 HCV-infected patients and 15 controls. TLR8 expression in PBMCs was significantly increased before treatment and recovered normal levels at week +48. Production of IL1b, IL6 and TNFα dependent on the activation of TLR8 in PBMCs was also increased in patients before DAA treatment, with a significant reduction at week +48. Combined expression of IFNγ and IL2 in CD4+ T cells in HCV-infected patients was significantly increased compared to controls and recovered normal levels at week +48. DAA-mediated clearance of HCV is associated with a decreased expression and activation of TLR8 in PBMCs until healthy control levels which is accompanied by a reduction in the Th1 response.

## 1. Introduction

Chronic hepatitis C virus (HCV) infection has been the leading cause of chronic liver disease, cirrhosis and liver transplant indication worldwide in the past decades [1,2]. Nevertheless, the appearance of effective interferon free direct acting anti-viral (DAA) treatment regimens has dramatically changed the natural history and epidemiology of HCV infection [3,4]. Progression from chronic hepatitis to different grades of fibrosis and cirrhosis may take several decades [5]. This progression is driven by an immune activation that entails a chronic low-grade inflammatory response that seems to be mediated at least in part by an interferon-driven innate immune response [6].

TLR7 and TLR8 recognize single-stranded RNA (ssRNA) in the forms of degradation products, nucleosides, and oligoribonucleotides [7]. Upon engagement of ssRNAs in endosomes, these antiviral sensors initiate the MyD88-dependent pathway, culminating in synthesis of type I and type III IFNs and proinflammatory mediators via activation of IRF7 and NF-κB, respectively, depending on the cell type [8]. Nevertheless, a recent study in HIV infected patients has proposed a role for adjuvant TLR8 stimulation, but not TLR7 or TLR9, in the reversion of latency in patient-derived latently infected CD4+ T cells, and a further promotion of T helper cell differentiation towards Th1 and Th17 [9]. Moreover, genetic variations in TLR8 have been associated with the clearance and progression of HCV infection with some gender influence [10].

The development of highly effective DAA treatments for HCV infection brings an excellent opportunity to assess the reversibility of the immune changes that take place over chronic infection. Thus, we aimed to analyze whether HCV eradication by DAA leads to a recovery in cell-mediated immune response and TLR8 expression and functionality.

## 2. Materials and Methods

### 2.1. Patients

Patients included in this study were drawn from a sample of chronically infected hepatitis C virus (HCV) candidates to initiate an interferon-free treatment regimen referred to our Hepatology Unit from 2015 to 2017. All patients were older than 18 years of age to be eligible and were included irrespective of the degree of liver fibrosis measured according to transient elastography (Fibroscan^®^; Echosense, Paris. France), HCV genotype, previous antiviral therapies, or response to those antiviral therapies. Exclusion criteria included human immunodeficiency virus co-infection or hepatitis B virus active infection, and current decompensated cirrhosis. Written informed consent was obtained from all patients and the study that was conducted conformed to the ethical guidelines of the Helsinki Declaration and had the approval of our local Ethics Committee.

### 2.2. Study Design

Prospectively, patients were included prior to interferon-free treatment initiation. Antiviral treatments and its duration were selected at the physician’s discretion based on the severity of liver fibrosis, HCV genotype, and the patient´s previous antiviral treatment response (Table 1). All patients had the same follow up every 4 weeks until the end of treatment (EOT) and from that point on, every 12 weeks until week +48 after EOT. Additionally, 15 healthy volunteers were included as a control group.

Blood samples were drawn after 12 h overnight fasting for flow cytometry analysis at two different time points: baseline and week +48 after EOT. Both baseline and at EOT, clinical, anthropometric, and analytical data were collected including complete blood count, liver and kidney function tests, soluble levels of total cholesterol, high-density lipoprotein (HDL)-cholesterol, triglycerides, glucose, and insulin. Insulin resistance was evaluated using the homeostasis model assessment (HOMA) [11].

### 2.3. TLR8 Expression in PBMCs

Intracellular expression of TLR8 was assessed on T cells, B cells, and monocytes by flow cytometry. Cells collected into sodium heparin tubes and peripheral blood mononuclear cells (PBMCS) were isolated by Ficol gradient and incubated with fluorochrome-conjugated anti-human CD3 (clone SK7), anti-human CD19 (clone HIB19), and anti-human CD14 (clone M5E2, all from BD Biosciences, San Diego, CA, USA) to identify T cells, B cells, and monocyte populations, respectively. To determine intracellular expression of TLR8, cells were fixed and permeabilized (BD Cytofix/Cytoperm, BD Biosciences) following manufacturer instructions and blocking with 2% pooled human serum for 20 min at 4 °C in the dark and intracellularly stained with the primary antibody (mouse anti-human anti-TLR8 (clone 44C143, Acris Antibodies, Herford, Germany) or mouse IgG2a isotype control antibody (eBioscience, San Diego, CA, USA) for 30 min followed by polyclonal goat-anti-mouse fluorescein isothiocyanate (FITC)-conjugated secondary antibody (Dako) for another 30 min. The cells were washed and acquired in FACS-Canto (BD Biosciences) cytometer. The expression of TLR8 was gated and analyzed (FACSDiva Software; BD Biosciences) as mean fluorescence intensity (MFI) with regard to the isotype control signal. See Figure S1A for the gating strategy and Figure S1B for backgating.

### 2.4. Assessment of TLR8 Function in Circulating PBMCs

Whole blood cells collected in sodium heparin tubes were polyclonally stimulated for 18 h with a specific agonist for human TLR8 (ssRNA 40, InvivoGen, San Diego, A, USA) at a final concentration of 1 ug/ml, in the presence or absence of brefeldin A (Sigma-Aldrich, St Louis, Missouri, USA) in polypropylene tubes, as previously described [12]. After culture, cells were stained with FITC-conjugated anti-human CD3, anti-human CD19, and anti-human CD14 (BD Biosciences, San Diego, CA, USA) to identify T cells, B cells, and monocyte populations, respectively. Then, red blood cells were lysed with FACS lysing solution (BD Biosciences) for 10 min. After being washed, the cells were permeabilized and intracellularly stained with phycoerythrin-labelled monoclonal antibodies against cytokines: interleukin (IL)-1β, tumor necrosis factor (TNF)-α, IL-6; and analyzed by flow cytometry using FACSDiva Software.

### 2.5. Production of IFNγ, IL2 and IL17 in T Cells

Intracellular cytokine staining was performed after polyclonal activation to detect the production of cytokines in the endoplasmic reticulum. PBMCs collected in sodium heparin tubes were stimulated for 4 hours with phorbol 12- myristate 13-acetate (PMA) (Sigma Aldrich, St Louis, Missouri, USA) and ionomycin (Calbiochem, Gibbstown, NJ, USA) in polystyrene tubes (lymphocytes) in the presence of brefeldin A (Sigma Aldrich).

After culture, cells were stained with APC-conjugated anti-CD4 (Clone SK3) antibody (BD Biosciences) to identify T lymphocytes. Thereafter, the red blood cells were lysed with FACS lysing solution (BD Biosciences), and the mononuclear cells were permeabilized using FACS Permeabilizing Solution (BD Biosciences) and intracellularly stained with FITC- or PE-conjugated cytokine-specific monoclonal antibodies (BD Biosciences) for IL-2, IFNγ and anti-IL-17 FITC (Clones: IL-2: 5344.11; IFNγ: 25723.11; IL-17: eBio64Dec17). BD Biosciences provided all the antibodies except Alexa Fluor 488-conjugated anti-IL-17 monoclonal antibody which was provided by eBiosciences.

### 2.6. Statistical Analysis

Data are expressed as the mean, standard error of mean (SEM), or median (interquartile range (IQR)) for quantitative data and counts, and percent for categorical data. As the present study was not designed to evaluate formal statistical hypotheses, no sample-size calculations were performed. Normally distributed values were analyzed by Student’s *t*-test, while Mann–Whitney test was used for non-parametric values. Wilcoxon test was used as nonparametric test for paired data. Categorical comparisons were performed using Χ2 or Fisher’s exact test, as appropriate. Statistical significance was considered when *p* < 0.05.

## 3. Results

### 3.1. Baseline Characteristics

Fifty-two chronically infected HCV patients (34 males (65.38%), mean age 53.00 years of age (±10.81)) met the inclusion criteria and were enrolled in the study. After baseline assessment, 2 patients lost their follow up and therefore 50 patients effectively initiated direct-acting antiviral treatment. Baseline anthropometrical, clinical, and analytical evaluation of the study population is summarized in Table 2. Overall, the study group is mainly composed of male naïve patients, in the sixth decade of life, with a mild liver disease due to a HCV genotype 1 prevailing over other genotypes. None of the patients included in the study were neither DAA therapy experienced nor cryoglobulinemic.

### 3.2. Evolution in the Expression profile of IFNγ, IL2, and IL17 in CD4+T Cells Pre and Post Direct-Acting Antiviral Therapy

**Figure 1:**
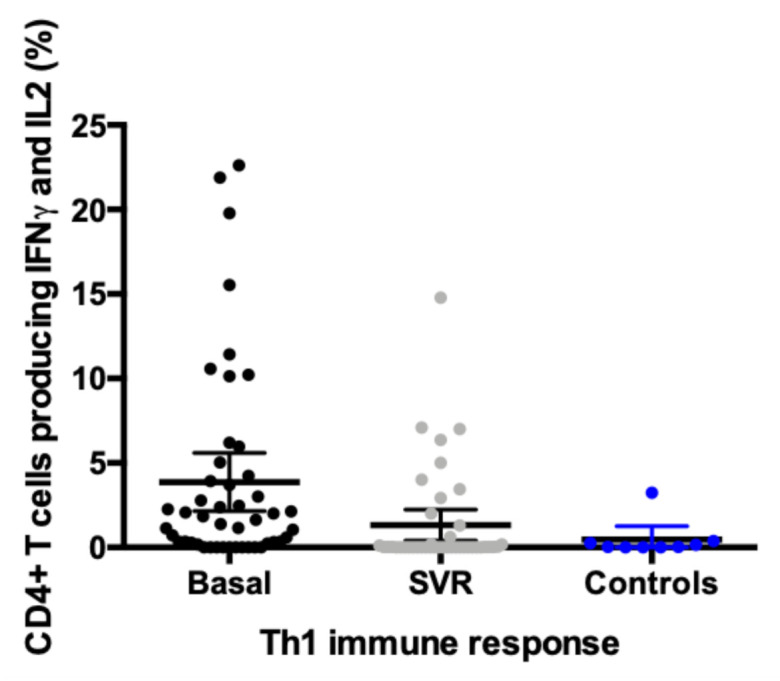
Distinctive Th1 immune response before and after treatment and compared to healthy controls. Intracellular cytokine staining in CD4+ T cells. Data represented as percentage of double positive IFN + IL2 expressed as mean (95% CI): basal expression 3.87 (2.15–5.59); SVR week +48 expression 1.32 (0.41–2.23); healthy controls expression 0.46 (0.00–1.27).

Pre-treatment, we found a distinctive higher expression profile of combined IFNγ and IL2 in CD4+ T cells in chronic HCV infected patients compared to healthy controls, pointing towards an increased Th1 immune response in this setting. Interestingly, this expression significantly diminishes after SVR at week +48, remaining the percentage indistinguishable to the one observed among controls (Figure 1). Both baseline increased expression of IFNγ and IL2 and subsequent decrement after SVR were observed irrespective of HCV genotype or liver fibrosis measured by transient elastography. None of these differences were observed regarding the expression of IL17 in CD4+ T cells (data not shown).

### 3.3. Distinctive Expression Profile of TLR8, Involved in the Recognition of Structural Components of Single Stranded-RNA Viruses

**Figure 2:**
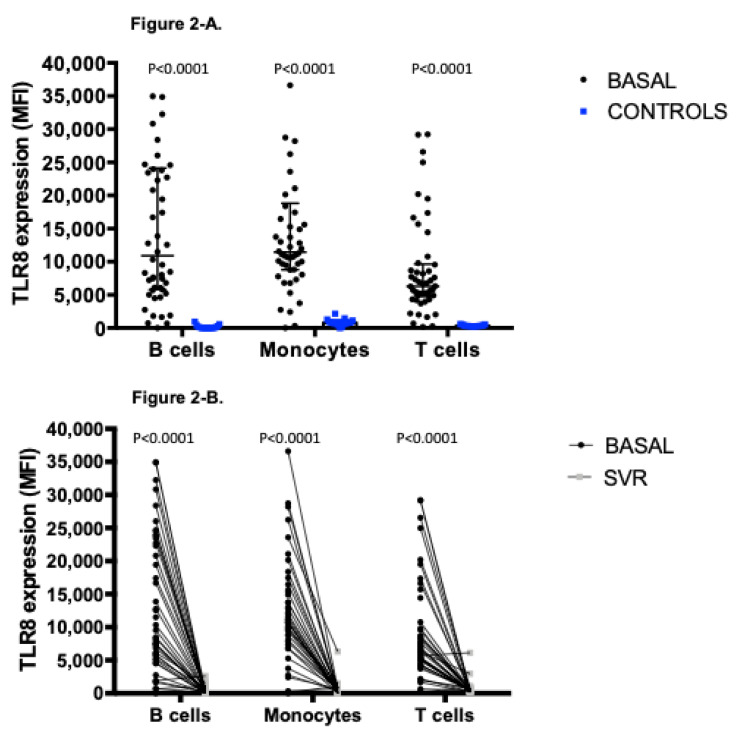
(**A**). TLR8 expression profile measured as mean fluorescence intensity (MFI) (median (interquartile range [IQR])) in B cells, monocytes, and T cells in hepatitis C infection (HCV) patients and healthy controls: TLR8 in B cells in HCV 10922.71 MFI (24115.27–5789.16) compared to controls 43.00 MFI (308.00–0.00); *p* < 0.0001. TLR8 in monocytes in HCV 11437.90 MFI (18826.14–8795.38) compared to controls 777.00 MFI (1120.00–636.00); *p* < 0.0001. TLR8 in T cells in HCV 6295.98 MFI (9634.35–4774.63) compared to controls 323.00 MFI (419.00–288.00); *p* < 0.0001). (**B**). TLR8 expression profile measured as mean fluorescence intensity (MFI) (median (IQR)) in B cells, monocytes, and T cells in HCV patients measured pre and post treatment: TLR8 in B cells pre-treatment 10922.71 MFI (24115.27–5789.16) compared to post-treatment 232.55 MFI (448.03–86.11); *p* < 0.0001. TLR8 in monocytes pre-treatment 11437.90 MFI (18826.14–8795.38) compared to post-treatment 595.94 MFI (826.78–159.58); *p* < 0.0001. TLR8 in T cells pre-treatment 6295.98 MFI (9634.35–4774.63) compared to post-treatment 411.47 MFI (627.94–285.83); *p* < 0.0001.

We found a significant higher expression profile of TLR8 in the three cell lines analyzed (B cells, T cells, and monocytes) in HCV infected patients compared to healthy controls (Figure 2A). This expression was found to be irrespective of gender, age, BMI, HCV genotype, or the severity of liver fibrosis. When we analyzed the expression of TLR8 at week +48 after EOT, we found that the expression in all cell subsets recovered levels indistinguishable from those found in healthy controls (Figure 2B). TLR8 expression in monocytes with respect to isotype control is shown in Figure S1C.

### 3.4. TLR8-Dependent Cytokine Expression Significantly Changes after Sustained Virological Response

**Figure 3:**
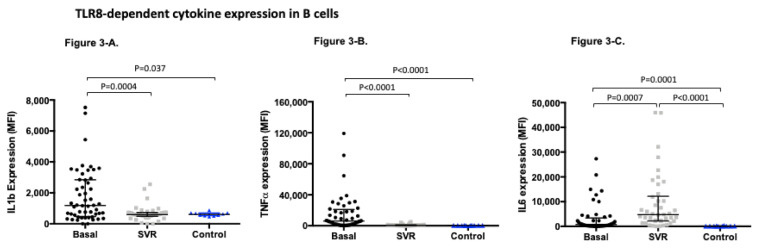
TLR8-dependent cytokine expression in B cells measured as mean fluorescence intensity (MFI) (median (IQR)) in HCV patients pre and post-treatment and healthy controls: (**A**). TLR8-dependant IL1b expression in B cells pre-treatment MFI (1181.0 (471.5–2845.0)) compared to post-treatment MFI (589.9 (483.1–736.4)) *p* = 0.0004; and compared to controls MFI (607.0 (523.6–702.7)) *p* = 0.037. (**B**). TLR8-dependant IL6 expression in B cells pre-treatment MFI (750.5 (207.2–3401.0)) compared to post-treatment MFI (4749.0 (2223.0–12196.0)) *p* = 0.0007; and compared to controls MFI (98.6 (80.9–142.3)) *p* = 0.0001. (**C**). TLR8-dependent TNF alfa expression in B cells pre-treatment MFI (6218.0 (1922.0–21013.0]) compared to post-treatment MFI (487.0 (286.8–1077.0)) *p* < 0.0001; and compared to controls MFI (399.4 (305.8–509.0)) *p* < 0.0001.

We analyzed the TLR8 dependent production of IL1b, IL6, and TNFα in B cells, T cells, and monocytes before treatment and at week +48 and compared it with the production obtained in healthy controls. The raw mean fluorescence intensity of IL1b, IL6, and TNFα in each subpopulation is shown in Figure S2. First, we assessed cytokine production in B cells where we observed that, in the case of IL1b and TNFα, both cytokines showed an increased expression pre-treatment that significantly decreased at week +48, recovering an expression profile comparable to controls. Nevertheless, in the case of IL6, the expression of this cytokine significantly increased at week +48 compared not only to baseline expression but also with the expression in the control group (Figure 3). This expression was found to be irrespective of gender, age, BMI, HCV genotype, or the severity of liver fibrosis.

**Figure 4:**
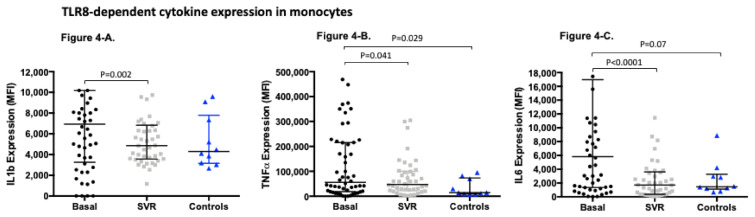
TLR8-dependent cytokine expression in monocytes measured as mean fluorescence intensity (MFI) (median (IQR)) in HCV patients pre and post-treatment and healthy controls: (**A**). TLR8-dependant IL1b expression in monocytes in HCV patients pre-treatment MFI (6937.0 (3254.0–10190.0)) compared to post-treatment MFI (4869.0 (3562.0–6835.0)) *p* = 0.002; and compared to controls MFI (4300.0 (3157.0–7793.0)) *p* = 0.189. (**B**). TLR8-dependant IL6 expression in monocytes in HCV patients pre-treatment MFI (5827.0 (1360.0–16971.0)) compared to post-treatment MFI (1718.0 (384.9–3609.0)) *p* < 0.0001; and compared to controls MFI (1468.0 (1119.0–3284.0)) *p* = 0.07. (**C**). TLR8-dependent TNF alfa expression in monocytes in HCV patients pre-treatment MFI (55740.0 (18550.0–215346.0)) compared to post-treatment MFI (47102.0 (22214.0–99991.0)) *p* = 0.041; and compared to controls MFI (14577.0 (9139.0–73250.0)) *p* = 0.029.

Second, we addressed the TLR8 dependent production of cytokines in monocytes (Figure 4). Interestingly, all three cytokines analyzed showed a significant decrease in their expression at week +48 recovering levels indistinguishable from those obtained in healthy controls. Notably, although there was a trend towards a higher expression of cytokines pre-treatment compared to the controls, these differences did not reach statistical significance in the case of IL6 and IL1b.

**Figure 5:**
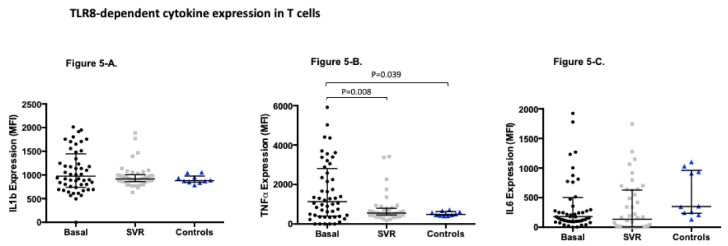
TLR8-dependent cytokine expression in T cells measured as mean fluorescence intensity (MFI) (median (IQR)) in HCV patients pre and post treatment and healthy controls: (**A**). TLR8-dependant IL1b expression in T cells in HCV patients pre-treatment MFI (974.2 (741.4–1448.0)) compared to post-treatment MFI (917.4 (859.5–1005.0)) *p* = 0,141; and compared to controls MFI (881.2 (846.6–976.6)) *p* = 0.254. (**B**). TLR8-dependant IL6 expression in T cells in HCV patients pre-treatment MFI (178.9 (102.1–502.1)) compared to post-treatment MFI (136.0 (4.79–629.8)) *p* = 0.513; and compared to controls MFI (351.0 (235.5–960.3)) *p* = 0.702. (**C**). TLR8-dependent TNF alfa expression in T cells in HCV patients pre-treatment MFI (1133.0 (400.0–2809.0)) compared to post-treatment MFI (552.9 (450.6–796.9)) *p* = 0.008; and compared to controls MFI (480.4 (427.6–625.3)) *p* = 0.039.

Finally, we analyzed the expression profile of cytokines in T cells (Figure 5). We observed that TNFα production decreased at week +48, reaching the same levels as observed in the controls. No differences were observed in terms of basal, post-treatment, and controls expression of IL1b and IL6 TLR8 dependent in T cells.

## 4. Discussion

Interaction between innate and adaptive immunity is required for the clearance of pathogens, such as HCV. Toll-like receptors are essential players in such an interaction, and ssRNA from HCV is supposed to stimulate TLR8 and induce the production of IFNγ. In the present study, we observed an increased expression of TLR8 not only in cells of the innate immune system such as CD14+ monocytes, but also in T and B cells from chronically HCV infected patients. In addition, these cells produced increased levels of inflammatory cytokines such as IL-1b, TNFα, and IL-6, after TLR8 in vitro stimulation. Very importantly, the overexpression and hyperactivation of TLR8 in chronic HCV infection was demonstrated for the first time to be reduced to levels comparable to healthy controls after 48 weeks of DAA treatment and SVR. In the present study, the activity of the TLR8 pathway seemed to correlate with the Th1 response, but not with Th17.

The expression of TLR in PBMC from HCV patients has been previously studied [13,14,15]. Most of them found increased activity of TLR2 and TLR4, including higher cytokine production following ex vivo TLR activation [13] with almost no involvement of other TLRs. Very few works have studied the expression of TLR8 in HCV infected patients and found equivalent results to ours [16,17]. We followed a different approach by measuring the protein expression by flow cytometry in the main three PBMC subsets (T, B, and monocytes), whereas the previous studies used qPCR. Such an overexpression of TLR8 associated with higher cytokine response could be explained by the incapacity of the innate and, secondary, the adaptative immune system, to clear the viral infection, as suggested by other authors [18]. Chen Yi Mei et al. found a decreased expression of TLR4 on monocytes and NK cells and a lower function of TLR4 and TLR7/8 in acute HCV infected subjects and after spontaneous clearance. In our study, the hyperexpression and function of TLR8 was associated with chronic infection but returned to healthy control levels after SVR.

Importantly enough, the upregulation of TLR8 was accompanied by an increased Th1 function as evidenced by IL-1 and IFNγ production but was not related to the Th17 response. It could be explained by a dependence on the interferon regulatory factor 5 (IRF5) but not the nuclear factor kappa B (NFkB) signaling to support a Th1 response [19]. However, the Th17 response would need both IRF5 and NFkB. In the case of HCV infection, despite both pathways being important for TLR8 activation, only the IRF5 could be inducing a Th1 response and the NFkB would be silenced. This is in agreement with the important type I interferon antiviral response in HCV infection [20]. On the other hand, it has been described that the Th17 response is not induced in chronic HCV infection but a T regulatory activity through the TLR2 pathway [21].

Our study has several limitations. First, we focused on TLR8 and not on other TLRs such as TLR2 and TLR4 that have been extensively involved in HCV infection. Second, we are aware of the lack of data about intracellular signaling that could explain the unbalance between Th1/Th17 responses, although it was out of the scope of the study.

In conclusion, this is the first study demonstrating a role for the TLR8 pathway in the sustained viral response induced by the new direct antiviral agents in HCV infection with a clear association with the Th1 response. The new therapy probably facilitates mounting an effective immune response against the HCV that is reflected by a restoration of the Th1 response to physiological levels.

## Figures and Tables

**Table 1:** Treatment specifications.

		N	%
**Treatment Regimen**			
	Ombitasvir+paritaprevir+ritonavir	5	10
	Ombitasvir+paritaprevir+ritonavir+dasabuvir	3	6
	Sofosbuvir+daclatasvir	13	26
	Sofosbuvir+ledipasvir	29	58
Ribavirin co-administered		3	6
**Treatment Duration**			
	8 weeks	8	16
	12 weeks	39	78
	24 weeks	3	6
**No Treatment Reasons**			
	Loss of follow up	2	4
Sustained virological response		50	100
Follow up week +48 assessment		43	86

**Table 2:** Basal clinical and analytical characteristics of the study population.

Baseline Study Group Characteristics			
Gender (male)		34	65.38
Age		53.00	10.81
BMI		26.89	6.29
Genotype			
	1a	17	32.69
	1b	12	23.08
	1	6	11.54
	3	13	25.00
	4	4	7.69
Previous antiviral treatment response			
	Naive	37	71.15
	Non-responder	8	15.38
	Relapser	2	3.85
	Adverse events	5	9.62
Fibrosis (measured by transient elastography)			
	F1	26	50.00
	F2	15	28.85
	F3	9	17.31
	F4	3	5.77
Leucocytes (103/µL)		6,00	1.71
Neutrophils (103/µL)		10.60	18.48
Haemoglobin (103/µL)		14.62	1.21
Platelets (103/µL)		195.58	60.43
INR		1.00	0.00
Prothrombin time (%)		82.35	10.48
Glucose (mg/dL)		105.80	46.55
Insulin		13.71	9.16
HOMA index		3.77	3.34
Creatinine (mg/dL)		1.06	0.47
Sodium (mg/dL)		140.28	2.32
ALT (U/L)		76.54	67.82
AST (U/L)		55.82	42.01
GGT (U/L)		84.86	115.80
Bilirrubin (mg/dL)		0.64	0.53
Albumin (mg/dL)		4.14	0.35
Triglycerides (mg/dL)		123.89	149.72
Total Cholesterol (mg/dL)		173.24	32.47
HDL-Cholesterol (mg/dL)		54.45	16.03
LDL-Cholesterol (mg/dL)		96.49	28.14
IgG (mg/dl)		1401.92	458.41
IgA (mg/dl)		238.82	124.34
IgM (mg/dl)		156.02	107.76

Data are expressed as the mean, standard error of mean (SEM) or median (interquartile range (IQR)) for quantitative data and counts, and percent for categorical data. BMI: body mass index; LDL: low-density lipoprotein; HDL: high-density lipoprotein; AST: aspartate aminotransferase; ALT: alanine aminotransferase; GGT: γ-glutamyl transpeptidase; HOMA: homeostasis model assessment.

## Data Availability

The datasets analyzed during the current study are available from the corresponding authors on reasonable request.

## Supplementary Materials

The following are available online at https://www.mdpi.com/article/10.3390/v13040635/s1 Figure S1A. Gating strategy; Figure S1B. Backgating; Figure S1C. TLR8 expression in Monocytes; Figure S2. IL-1b, TNF-a and IL-6 expression after TLR8 agonist treatment in different subpopulations
