# Supplementary material for: Successful Direct Acting Antiviral Therapy in Chronic Hepatitis C Normalizes IFNγ and IL2 Production in T Cells Together with TLR8 Expression and Functionality in Peripheral Blood Mononuclear Cells
Source: Viruses. 2021 Apr 7;13(4):635. doi: 10.3390/v13040635 (PMC8068025; doi:10.3390/v13040635)
Supplement: Supplementary file 1 [file viruses-13-00635-s001.pdf]

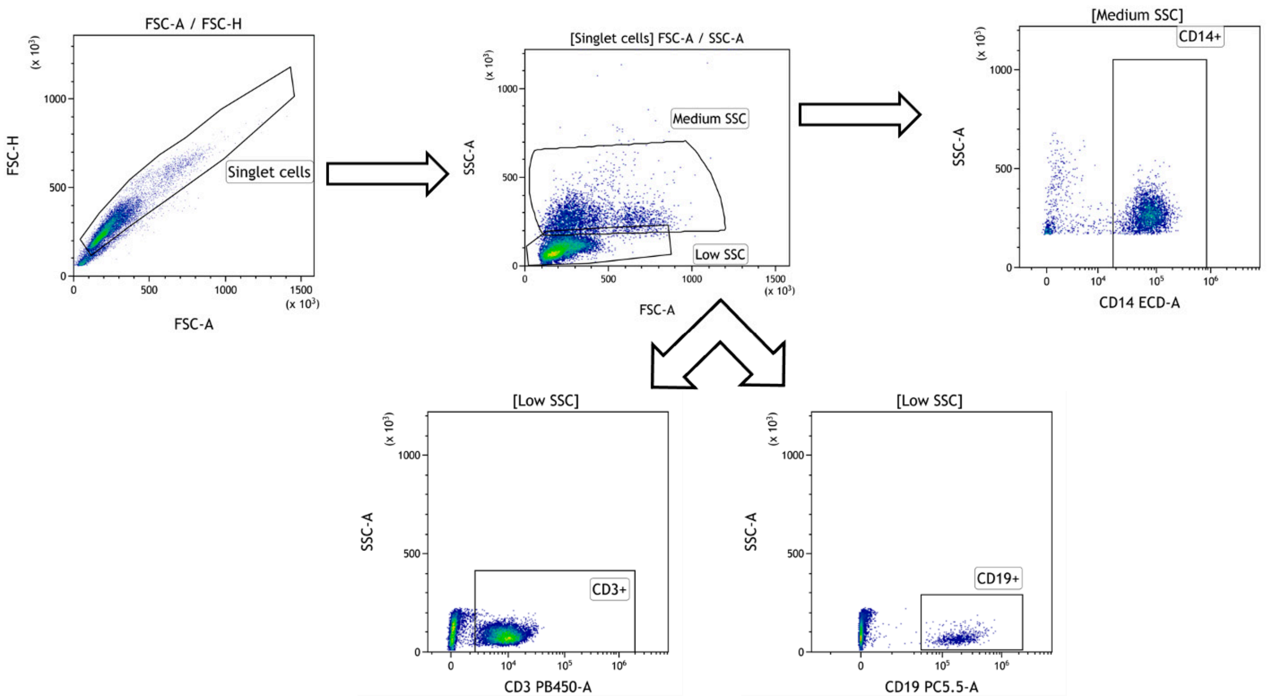

Supplementary Figure 1A. Gating strategy. The peripheral blood mononuclear cells were selected as singlet cell by forward-scatter channel (FSC)-Area (A) and -High (H). Subsequent gating in medium side-scatter channel (SSC) and low SSC was performed. Within medium-SSC, the monocytes were identified with anti-CD14-ECD, whereas within low-SSC, T and B lymphocytes were selected with anti-CD3 and anti-CD19, respectively.

Supp Figure 1B

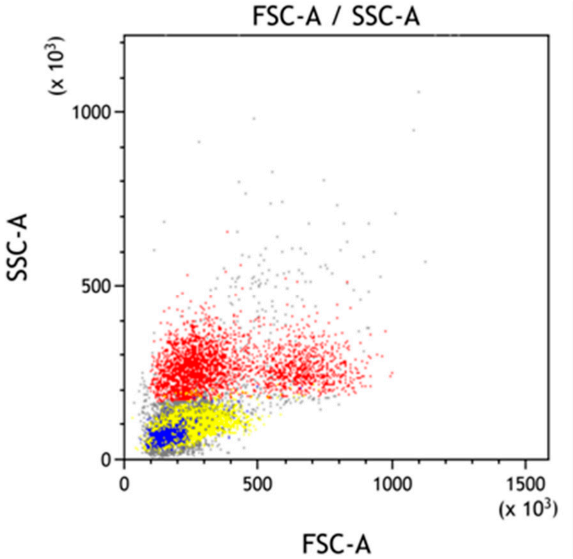

Supplementary Figure 1B. Backgating. After backgating, the subpopulations are depicted as Monocytes in red, T-lymphocytes in yellow, and B-lymphocytes in blue.

Supp Figure 1C

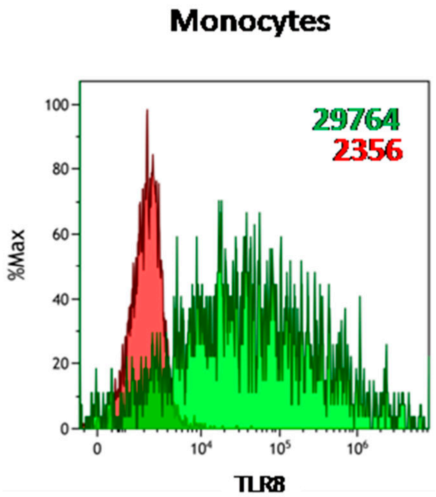

Supplementary Figure 1C. TLR8 expression in Monocytes. The monocytes were identified following gating strategy (Supplementary Figure 1A) and mean fluorescence intensity (MFI) of TLR8 expression (green) and isotype control (red) was measured.

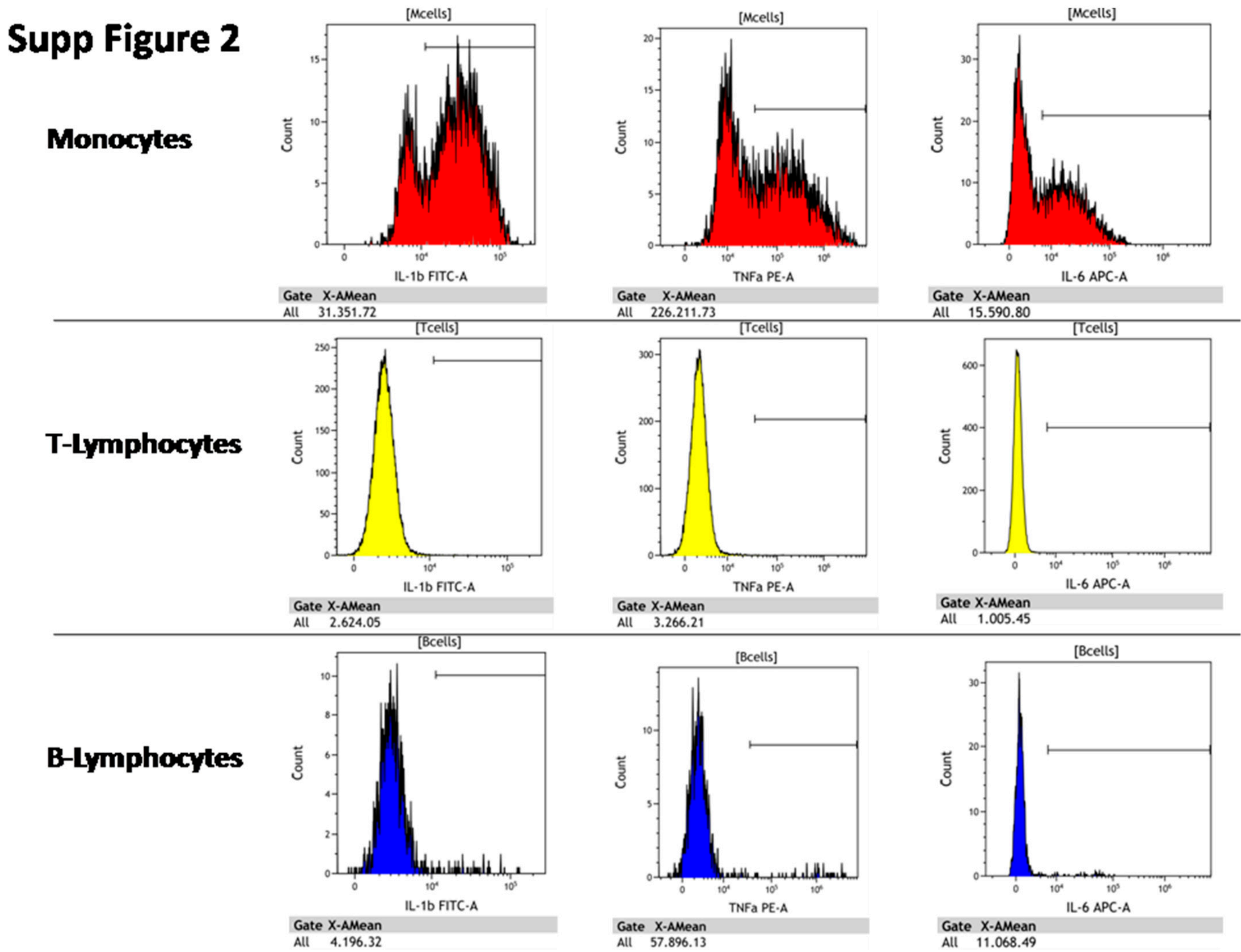

Supplementary Figure 2. IL-1b, TNF-a, and IL-6 expression after TLR8 agonist treatment in different subpopulations. The raw mean fluorescence intensity in each subpopulation is shown. After 18 hours of culture with single-stranded RNA (ssRNA) 40 (TLR8 agonist) the cells were stained with monoclonal antibodies to identify monocytes (red), T (yellow), and B lymphocytes (blue) with CD14, CD3, and CD19, respectively. Further permeabilization and anti- interleukin-1b (IL-1b, right panels), tumor necrosis factor (TNF)-α in center panels, and IL-6 (on the left panels) were used to measure the expression of soluble factors.
